# Fabrication of poly(3-hydroxybutyrate-co-3-hydroxyhexanoate) Fibers Using Centrifugal Fiber Spinning: Structure, Properties and Application Potential

**DOI:** 10.3390/polym15051181

**Published:** 2023-02-26

**Authors:** Chris Vanheusden, Jan Vanminsel, Naveen Reddy, Pieter Samyn, Jan D’Haen, Roos Peeters, Anitha Ethirajan, Mieke Buntinx

**Affiliations:** 1 Materials and Packaging Research & Services, Institute for Materials Research (IMO-IMOMEC), Hasselt University, Wetenschapspark 27, 3590 Diepenbeek, Belgium; 2 SIRRIS, Department Circular Economy and Renewable Materials, Gaston Geenslaan 8, 3001 Leuven, Belgium; 3 Analytical & Microscopical Services, Institute for Materials Research (IMO-IMOMEC), Hasselt University, Wetenschapspark 1, 3590 Diepenbeek, Belgium; 4 Nano-Biophysics and Soft Matter Interfaces (NSI) Group, Institute for Materials Research (IMO-IMOMEC), Hasselt University, Wetenschapspark 1, 3590 Diepenbeek, Belgium

**Keywords:** polyhydroxyalkanoates, poly(3-hydroxybutyrate-co-3-hydroxyhexanoate), centrifugal fiber spinning, fiber morphology, fiber annealing, top-layer

## Abstract

Biobased and biodegradable polyhydroxyalkanoates (PHAs) are currently gaining momentum. Poly(3-hydroxybutyrate-co-3-hydroxyhexanoate) (PHBHHx) polymer has a useful processing window for extrusion and injection molding of packaging, agricultural and fishery applications with required flexibility. Processing PHBHHx into fibers using electrospinning or centrifugal fiber spinning (CFS) can further broaden the application area, although CFS remains rather unexplored. In this study, PHBHHx fibers are centrifugally spun from 4–12 wt.% polymer/chloroform solutions. Beads and beads-on-a-string (BOAS) fibrous structures with an average diameter (ϕ_av_) between 0.5 and 1.6 µm form at 4–8 wt.% polymer concentrations, while more continuous fibers (ϕ_av_ = 3.6–4.6 µm) with few beads form at 10–12 wt.% polymer concentrations. This change is correlated with increased solution viscosity and enhanced mechanical properties of the fiber mats (strength, stiffness and elongation values range between 1.2–9.4 MPa, 11–93 MPa, and 102–188%, respectively), though the crystallinity degree of the fibers remains constant (33.0–34.3%). In addition, PHBHHx fibers are shown to anneal at 160 °C in a hot press into 10–20 µm compact top-layers on PHBHHx film substrates. We conclude that CFS is a promising novel processing technique for the production of PHBHHx fibers with tunable morphology and properties. Subsequent thermal post-processing as a barrier or active substrate top-layer offers new application potential.

## 1. Introduction

Plastics are ubiquitous due to their high versatility, low weight and low cost with a variety of applications in packaging, building, automotive, electronics and other industries. However, due to increasing environmental awareness, the depletion of fossil fuels and increasing plastic pollution, bioplastics are currently gaining momentum. The bioplastics market still represents less than 1% share of global plastic production, but is estimated to increase from around 2.2 million tons in 2022 to approximately 6.3 million tons in 2027 [1]. Bioplastics have several advantages over petroleum-based plastics such as a lower carbon footprint, energy efficiency and/or biodegradability or compostability [2]. However, bioplastics often exhibit inferior properties such as thermal instability, low melt strength and poor processability that can limit their application [3]. In addition, current challenges of bioplastics include their cost, large-scale production, recycling and legislation [2,4,5]. The most widely used bioplastics in the global market include non-biodegradable polymers such as polyethylene terephthalate (bio-PET), polyethylene (bio-PE), and polypropylene (bio-PP) and biodegradable polymers such as polybutylene adipate terephthalate (PBAT), polybutylene succinate (PBS), polylactic acid (PLA), starch blends and polyhydroxyalkanoates (PHAs) [1]. Among these bioplastics, PHAs have attracted considerable attention as alternatives to petroleum-based plastics [6]. These biopolymers can be synthesized by bacteria from a wide range of carbon-rich substrates such as fats and sugars, but more recently from a variety of waste streams and industrial byproducts [7]. PHAs show promise for use in food packaging applications with suitable barrier and mechanical properties, as well as heat resistance [8]. PHAs also appear to be excellent contenders among bioplastics in terms of environmental load, versatility, and integration possibilities in current waste management systems [9]. Despite their wide application potential, high production costs and processing challenges still limit competition with conventional plastics [10]. Poly(3-hydroxybutyrate) (PHB), poly(3-hydroxybutyrate-co-3-hydroxyvalerate) (PHBV), and poly(3-hydroxybutyrate-co-3-hydroxyhexanoate) (PHBHHx) are widely investigated PHA family members [8]. Medium chain length (mcl) PHAs such as PHBHHx show more promise in flexible applications [11], while short chain length (scl) PHAs such as PHB and PHBV are preferred for rigid applications. PHBHHx shows improved thermal stability compared to PHB and PHBV [12] and a suitable processing window for extrusion and injection molding, but can still thermally degrade under the influence of high temperatures and increased shear during twin screw extrusion [11]. More efforts should be made to increase the use of PHBHHx in packaging applications, such as multilayer films and active packaging [13]. Therefore, investigating novel processing techniques, such as fiber spinning, are important for further broadening the application potential of PHBHHx.

The processing of PHBHHx into fibers can be a valuable approach to create and design more applications and to add specific functional properties. Micro- and nano-sized fibers have gained significant attention for use in the biomedical sector in tissue engineering, drug delivery or biosensing [14]. These fibers can also be applied in food packaging systems [15]. Electrospinning (ES) is the most widely used technique for the production of micro- and nanofibers with applications in diverse fields [16]. A variety of synthetic and natural polymers have been electrospun into fibers [17]. In addition, electrospun fibers fabricated from PHAs [18] such as PHB [19], PHBV [20,21,22,23], PHBHHx [24,25,26,27,28,29,30], poly(3-hydroxybutyrate-co-4-hydroxybutyrate) (P3HB4HB) [31] and blends thereof [32] have been reported in the literature. After the spinning process, fibers can undergo several thermal posttreatments, including annealing and heat pressing, to produce transparent or translucent films or multilayer structures with excellent mechanical, barrier, and optical properties for food packaging applications [33]. Several attempts have been made to develop continuous electrospun-based films for biopolymers, including PHB [34], PHBV [35,36,37], and PLA [38]. Electrospun multilayer structures including paper/PHB and PLA [39], and nanocellulose/PHB and PHBV have also been reported [40]. These electrospun continuous films or multilayer structures show enhanced mechanical and/or barrier properties. Electrospinning has also been used to encapsulate active ingredients to obtain degradable packaging membranes with functional properties such as a high gas barrier and antimicrobial effects [41,42]. Despite the versatility of ES, its industrial use is limited because of low production speed, high cost per gram of fiber, and the need for high voltages [43].

On the other hand, centrifugal fiber spinning (CFS) is considered as a more promising method than ES for the fabrication of fibers due to its simplicity, high rate of fiber production and ability to produce continuous fibers from polymer solutions into enhanced nonwoven structures [44]. The fiber morphology is mainly determined by the intrinsic properties of the polymer solution (concentration and molecular weight) together with the operational parameters (centrifugal speed, nozzle diameter and collector distance) [45]. Only a limited number of studies were previously reported on CFS of PHAs (without fillers or additives), such as PHB [46,47] and PHBV [48]. Upson et al. [48] showed that the morphology of PHBV fibers depends on the solution viscosity, with the formation of more continuous fibers at higher polymer concentrations (20 and 25 *w*/*v*%). Despite these results, they showed that under specific processing conditions, continuous PHBV fibers were too brittle to properly characterize for mechanical performance. In addition, the increased ductility of the PHBV fiber mats was at the expense of strength and stiffness. Therefore, using PHBHHx to fabricate continuous fibers with sufficient tensile strength and stiffness, while maintaining desired flexibility (ductility) via CFS could possibly increase the use of these materials for a wide range of applications. Moreover, the processing of PHBHHx into fibers with CFS seems more promising compared to ES, because of higher production rates and better scalability. The fact that centrifugally spun fiber mats are often loosely packed, compared to the densely-packed electrospun fiber mats [43], can make the processing of these fibers into continuous films or top-layers a bit more challenging. To the best of our knowledge, no studies were previously performed on the production and thorough characterization of PHBHHx fibers with the CFS technique and the post-processing of these centrifugally spun fiber mats into films, multilayer or top-layer structures.

The aim of this study is to investigate structure-property relationships of centrifugally spun PHBHHx fibers. This article focuses on the effects of the PHBHHx concentration (in chloroform (CHCl_3_)) on rheology, morphology, chemical, thermal and mechanical properties of the centrifugally spun fibers. In addition, the feasibility to deposit and process the obtained centrifugally spun PHBHHx fiber mats as top-layers on substrates via post-thermal treatment is investigated.

## 2. Materials and Methods

### 2.1. Materials

PHBHHx pellets (KANEKA Biodegradable Polymer Green Planet™) containing 10.5 mol% 3HHx were kindly provided by Kaneka (Westerlo-Oevel, Belgium). PHBHHx has a weight-average molecular weight (M_w_), number-average molecular weight (M_n_), and polydispersity index (PDI) of 3.3 × 10^5^ g/mol, 1.2 × 10^5^ g/mol, and 2.7, respectively, as measured by gel permeation chromatography (GPC). Chloroform (CHCl_3_, AnalaR NORMAPUR) was purchased from VWR Chemicals (Leuven, Belgium) and was used without further purification.

### 2.2. Centrifugal Fiber Spinning (CFS)

Different concentrations of polymer solutions (2–14 wt.%) were prepared by adding PHBHHx to chloroform under magnetic stirring for 1 h at 55 °C in sealed glass vials until all polymer dissolved, with $wt.\%=\left(m_{PHBHHx}/\left(m_{PHBHHx}+m_{chloroform}\right)\right)\times 100$. The solution was cooled to room temperature prior to spinning. A custom-built CFS setup [49,50,51,52] with an aluminum arm-style spinneret and two aluminum nozzles was used to produce PHBHHx fibers. The fibers were spun at a spinneret speed of 4000 rpm, with a nozzle diameter of 0.6 mm and a collector distance of ± 10–12 cm. The processing conditions and polymer concentration range were selected based on previous studies with the custom-built CFS equipment [49,50,51,52,53]. In addition, the collector distance and rotational speed were set with respect to the limits of the equipment and were maintained constant throughout the experiments to investigate the transition from beads to continuous fibers by changing the concentration of the polymer solution. After reaching and maintaining a rotational speed of 4000 rpm, the solutions were added via a syringe pump to the center of the rotating spinneret to ensure a continuous liquid flow. Fiber spinning experiments were performed at room temperature under a fume hood. After spinning, fibers were collected using a homemade fork for characterization and further processing.

### 2.3. Characterization of PHBHHx Solutions, Fibers and Films

#### 2.3.1. Rheological Characterization

The shear viscosity of the PHBHHx/CHCl_3_ solutions was measured using an ARG2 stress-controlled rheometer (TA Instruments, New Castle, United States) with cone-and-plate geometry (40 mm diameter, 2° cone angle). Measurements were performed at 25 °C using a solvent trap to reduce solvent evaporation. Solutions were loaded under a static geometry, followed by a pre-shear of 10 s^−1^ for 30 s and an equilibration step for 60 s. Viscosity was recorded for shear rates ranging from 0.1 to 1200 s^−1^ with a steady-state tolerance of 3%. The zero-shear viscosity ($\eta_{0}$) was calculated in the shear rate range of $\dot{\gamma }$ = 1 to 1000 s^−1^ via the Cross model [54]:

(1) $$\eta_{eff}=\eta_{\infty }+\frac{\eta_{0}-\eta_{\infty }}{1+{\left(k\dot{\gamma }\right)}^{n}}$$

and for $\eta_{0}>>\eta_{\infty }$ Equation (1) simplifies to

(2) $$\eta_{eff}\left(\dot{\gamma }\right)=\eta_{0}{m\left(\dot{\gamma }\right)}^{n-1}$$

with $\eta_{eff}\left(\dot{\gamma }\right)$ as the effective viscosity measured as a function of shear rate $\dot{\gamma }$, $\eta_{0}$ as the zero-shear viscosity, $\eta_{\infty }$ as the infinite shear viscosity and k and n being the fitting coefficients. The specific viscosity represents the increase in viscosity that is contributed by the polymer in solution and was calculated for comparison purposes with the following equation [55]:

(3) $$\eta_{sp}=\frac{\eta_{0}-\eta_{s}}{\eta_{s}}$$

with $\eta_{sp}$ as the specific viscosity and $\eta_{s}$ as the solvent viscosity [56].

#### 2.3.2. Scanning Electron Microscopy (SEM)

The morphology of the fibers and films was analyzed via SEM images acquired using a Zeiss 450 FEGSEM with Gemini 2 optics (ZEISS, Zaventem, Belgium) at 10 kV under high vacuum. The fibers and films were sputtered with a thin layer of gold-palladium before analysis to reduce charging. The distribution in fiber diameters was measured from SEM images using ImageJ software (Maryland, United States). At least 132 fibers were measured per sample type and evenly divided over two to four SEM images (depending on the fiber diameter).

#### 2.3.3. Thermal Properties

The melting and crystallization behavior of the PHBHHx materials was analyzed with differential scanning calorimetry (DSC) under an inert atmosphere (50 mL/min N_2_) using a Q200 instrument (TA Instruments, New Castle, United States). Fiber mat samples of approximately 4–6 mg and film samples of about 6–7 mg in sealed aluminum pans were heated from −30 °C to 160 °C before being kept isothermal for 2 min. The samples were then cooled to −30 °C and kept constant for 2 min before heating to 160 °C. The heating and cooling rates were set at 20 °C/min. The crystallinity (X_c_) of the samples was calculated using the following equation [57]:

(4) $$X_{c}=\frac{∆H_{m}}{∆H_{m}^{0}}\times 100$$

with $∆H_{m}$ as the melting enthalpy derived from the heating cycle and $∆H_{m}^{0}$ as the melting enthalpy of the 100% crystalline polymer (115 J/g [58,59]). The crystallization enthalpy ΔH_c_ was derived from the first cooling cycle.

#### 2.3.4. Mechanical Properties

**Figure 1:**
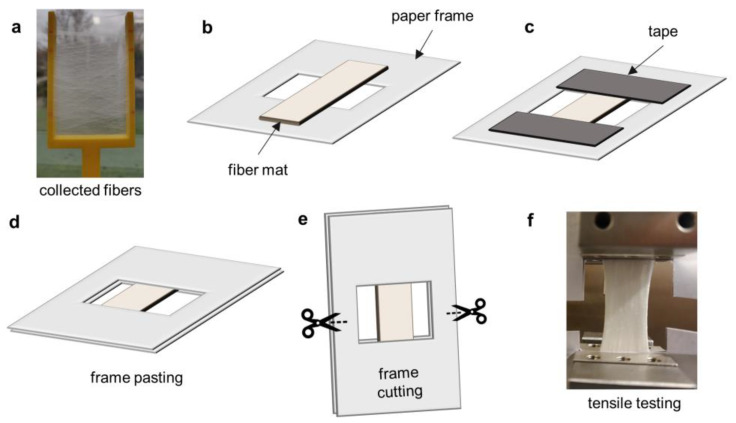
Schematic of the tensile testing set-up of PHBHHx fiber mats. Manual collection of fiber mat with a homemade fork (**a**), placement of fiber mat on a paper frame (**b**), taping of fiber mat onto paper frame (**c**), pasting of fiber mat between paper frames (**d**), cutting of paper frame prior to tensile testing (**e**) and example of fiber mat during tensile testing (**f**).

The mechanical properties of the PHBHHx fiber mats were determined by tensile testing using a 5ST universal testing machine (Tinius Olsen, Redhill, United Kingdom). The tensile testing set-up is shown in Figure 1. The fiber mats with a width of ±1–2 cm were cut to a length of 6 cm, weighed, and pasted between two paper frames. This sample preparation avoids fiber mat damage and slipping at the grips, with improved sample positioning in the clamps. The paper frame was cut prior to tensile testing. The cross-sectional area of the fiber mats was determined using the fiber mat weight, length and polymer density (ρ = 0.00119 g/mm^3^) following a similar method as described in [60]. The raw tensile data were normalized to stress-strain curves with the following equation:

(5) $$\sigma =\frac{F}{A}=\rho \frac{F}{m}L$$

with σ as the tensile stress (MPa), F as the recorded force (N), A as the fiber mat cross-sectional area, m as the fiber mat weight and L as the fiber mat length. Measurements were performed at a crosshead speed of 10 mm/min, clamping distance of 20 mm, pre-load of 0.1 N and pre-load speed of 1 mm/min. The tensile strength (σ) was calculated as the peak stress, and Young’s modulus (E) was calculated from the linear slope at low strains (3–5%). The elongation at break (ε) was determined as the strain (%) at 95% peak load drop. The fiber mats were conditioned at 23 °C and 50% relative humidity (RH) for at least 3 days prior to tensile testing. The mechanical properties are reported as the average of 10 measurements on samples from two distinct fiber mat batches.

### 2.4. Deposition of Fiber Mats on PHBHHx Films

As a proof of concept, PHBHHx fiber mats produced from 10 wt.% solutions by CFS were post-processed into continuous films. For this, 1 g of PHBHHx fiber mats were compression molded using a hot-press PCH-600DG (Henan Chuanghe Laboratory Equipment Co. Ltd., Zhengzhou, China) to obtain thin films with a thickness of ±100 µm. The fibers were preheated for 4 min at 145 °C without pressure, followed by pressure cycles of 3 and 15 MPa at 145 °C (both for 2 min) and water cooling for 10 min at 5 MPa pressure.

To validate the use of PHBHHx fibers as continuous top-layers on substrates, lower amounts of fiber mats were deposited on PHBHHx substrates, as described as follows. First, PHBHHx substrate films (10 cm × 10 cm) of ±160–170 µm thickness were fabricated via compression molding using the hot-press. PHBHHx pellets were dried for ±3 days at 65 °C and approximately 2.1 g was placed in a stainless-steel mold, sandwiched between Teflon sheets and aluminum plates and compressed using the above settings. Secondly, small amounts of PHBHHx fiber mats (±0.01–0.03 g), centrifugally spun from 10 wt.% solutions, were placed onto PHBHHx film substrates (cut to ±2.5 cm × 2.5 cm) and both materials were dried at 65 °C for 24 h in an opened petri-dish. The thin fiber layers were then attached to the virgin substrates by annealing in the hot press for 6 min at 160 °C (upper plate) and 40 °C (lower plate) without pressure. The resulting films were cooled to room temperature.

## 3. Results and Discussion

### 3.1. PHBHHx Fiber Morphology

**Figure 2:**
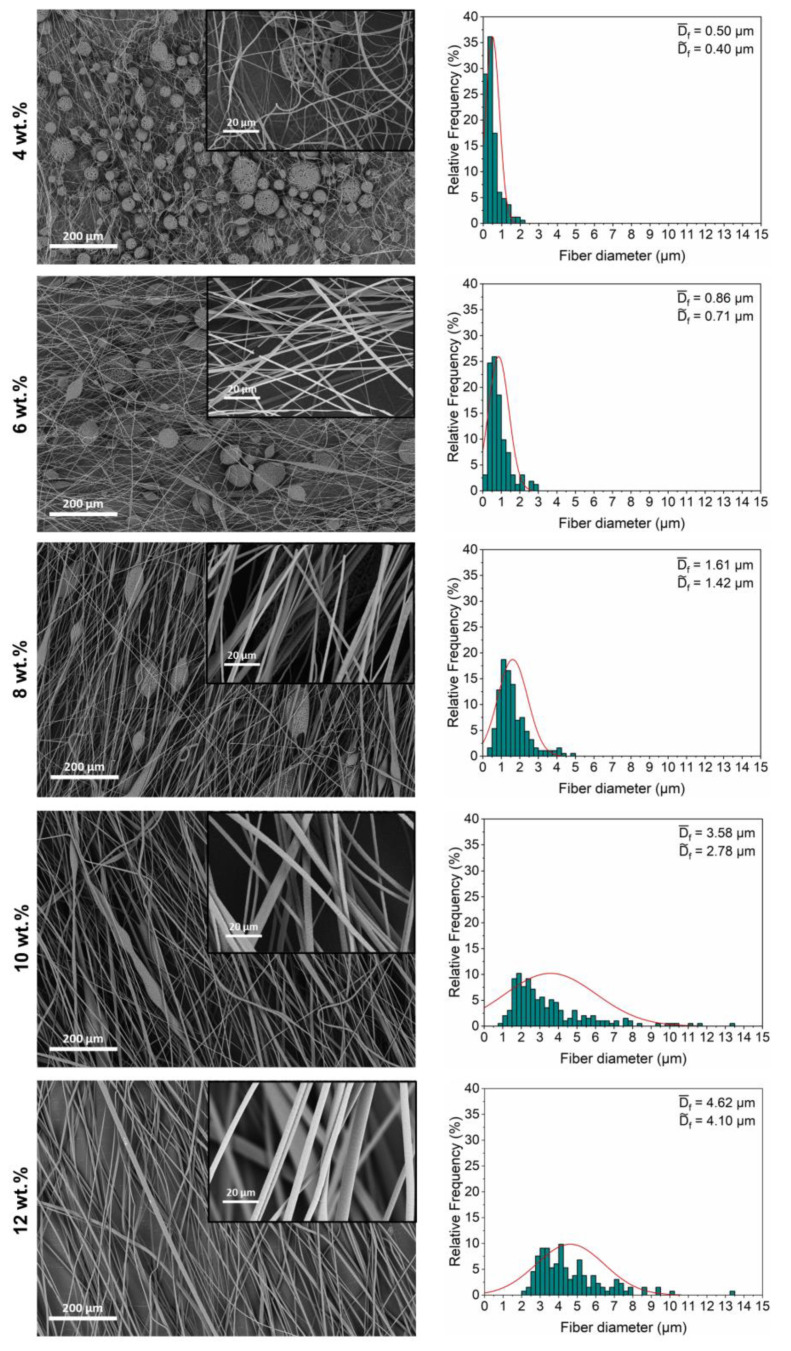
SEM images and corresponding diameter distributions (average (${\overset{-}{D}}_{f}$) and median (${\tilde{D}}_{f}$ ) fiber diameter) of PHBHHx fibers spun from 4, 6, 8, 10 and 12 wt.% polymer concentrations.

After the centrifugal spinning of PHBHHx fibers from solutions with different concentrations, SEM images of the fibers were analyzed. The associated fiber diameter distributions for 4–12 wt.% polymer solutions are shown in Figure 2. Beads and beaded fibers or beads-on-a-string (BOAS) fibrous structures form at low polymer concentrations (4–8 wt.% PHBHHx) whereas more continuous fibers with few beads form at higher polymer concentrations (10–12 wt.% PHBHHx).

At polymer concentrations below 4 wt.%, the fiber production rate is very low and results in a beaded morphology (data not shown). Fibers spun from 4 wt.% solutions are characterized by submicron average diameters (${\overset{-}{D}}_{f}$ = 0.50 ± 0.37 µm) with nearly spherical and porous beaded structures. These beads are formed due to the breakdown of solution jets into droplets at low polymer concentrations, i.e., at lower solution viscosity. The surface tension tries to minimize the jet surface area by forming small droplets with a spherical geometry. Complementary, the breakup of the jet into droplets by the surface tension driven Rayleigh instabilities cannot be prevented due to a low visco-elastic force in low polymer concentration solutions, resulting in spherical bead formation [61]. Alongside, bead porosity is formed by the collapse of a thin polymer shell at the surface of a solution drop, formed by rapid solvent evaporation [62]. The visco-elastic forces are sufficient to counteract the jet breakup and the bead shape changes from spherical-like to a BOAS morphology when the polymer concentration is increased to 6 wt.%, which is associated with an increase in the average fiber diameter to ${\overset{-}{D}}_{f}$ = 0.86 ± 0.55 µm. The elongation of beads into BOAS morphology is even more pronounced at a polymer concentration of 8 wt.%, with an increased average fiber diameter of ${\overset{-}{D}}_{f}$ = 1.61 ± 0.81 µm. A steep increase in fiber diameter together with the formation of more continuous fibers are apparent above polymer concentrations of 8 wt.%. The average fiber diameters (${\overset{-}{D}}_{f}$) at polymer concentrations of 10 and 12 wt.% increase to respectively 3.58 ± 2.46 µm and 4.62 ± 1.84 µm due to less stretching (thinning) of the polymer jet with higher solution viscosity. The transition to continuous fibers with increased diameters is also associated with a broadening of the diameter distribution, as previously reported for centrifugally spun PEO [63]. An increased polymer concentration also affects the skewness of the distribution, which is similarly observed for centrifugally spun PA6 [64].

The fibers are rather randomly oriented at low PHBHHx concentrations (4–6 wt.%), while more orientation seems apparent at higher concentrations (≥8 wt.%). This could be attributed to the higher production rate during spinning. Tong et al. also showed a higher degree of fiber alignment in PHBV mats at higher polymer concentrations during electrospinning which was explained by a shift of the traveling speed profile to a low speed region due to an increase of the jet mass [65]. This can also be observed during CFS because thinner and lighter fibers undergo more air drag, pushing them around with higher speed to lose their alignment. Further, dissolving PHBHHx in CHCl_3_ above 12 wt.% is feasible and results in continuous fibers. However, the production of fibers above concentrations of 12 wt.% is practically more challenging due to extra stirring of the solution and more complicated flow of the viscous solution in the delivery syringe and spinneret. Therefore, the fiber spinning of PHBHHx is described for polymer concentrations until 12 wt.%.

From the SEM studies, it is clear that PHBHHx can be centrifugally spun into fibers with different morphologies and fiber diameters under a concentration dependent processing window. Higher polymer concentrations facilitate the formation of more smooth, continuous and thicker fibers (Figure 2).

### 3.2. Rheological Characterization of PHBHHx/Chloroform Solutions

**Figure 3:**
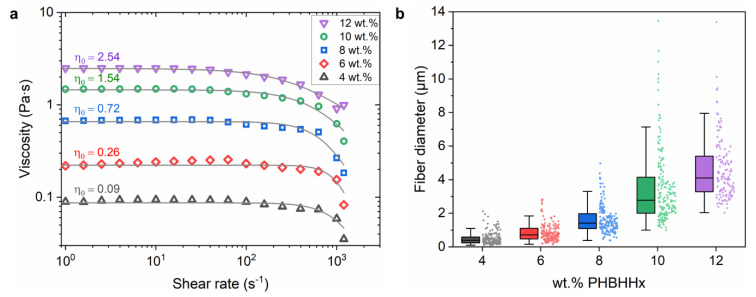
(**a**) Steady-shear viscosity as a function of shear rate for different PHBHHx/chloroform solutions showing shear-thinning behavior. The Cross model fits are shown as solid lines together with the corresponding zero-shear viscosity $\eta_{0}$ (*n* = 2). (**b**) Diameter of PHBHHx fibers spun with different polymer concentrations. The box is determined by the 25th and 75th percentiles together with the median fiber diameter (horizontal solid line), and the error bars show the range within 1.5 of the IQR (interquartile range).

To explain the relation between the polymer concentration and the respective CFS fiber morphology, viscosity measurements were performed by rotational rheometry. In Figure 3a, the steady shear viscosity ($\eta_{eff}$) (Pa·s) is plotted as a function of shear rate (s^−1^) for 4–12 wt.% PHBHHx/chloroform solutions. Shear-thinning behavior is observed for all concentrations, with a Newtonian plateau at lower shear rates. This shear thinning behavior is fitted with the Cross model (see solid lines in Figure 3a) and the zero-shear viscosity $\eta_{0}$ is calculated. The viscosity of the solutions increases with polymer concentration due to polymer chain entanglement. The zero-shear viscosity ($\eta_{0}$) of the solutions (4–12 wt.%) ranges between 0.09 and 2.54 Pa·s, corresponding to specific viscosity values ($\eta_{sp}$) in the range of 159 to 4740. In solutions with higher polymer concentrations, the viscoelastic forces are sufficient to counteract capillary breakup and bead formation. Due to a decrease in beads, more bulk polymers remain in the fiber which further increases the fiber diameter with polymer concentration [49]. In addition, a higher viscosity also prevents the elongation of the polymer jet, resulting in larger fiber diameters (Figure 3b) [66]. Such an increase in fiber diameter with polymer concentration and viscosity has been observed for a wide range of polymeric materials for CFS and ES [43,44,67,68,69,70].

**Figure 4:**
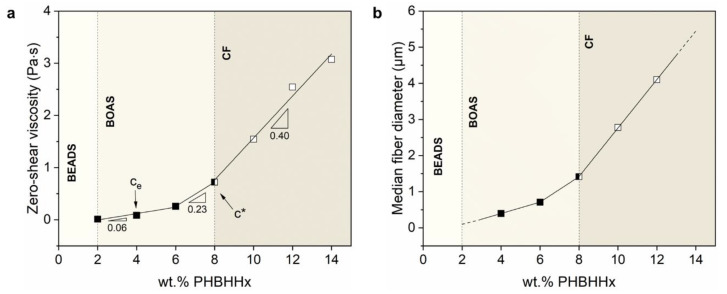
(**a**) Zero-shear viscosity of 2–14 wt.% PHBHHx/chloroform solutions with distinct regions of chain entanglement and beads, beads-on-a-string (BOAS) and continuous fibers (CF) and (**b**) median fiber diameter for 4–12 wt.% PHBHHx fibers.

The zero-shear viscosity and median fiber diameter are studied as a function of polymer concentration to determine the characteristic chain entanglement concentrations, which are essential for proper fiber fabrication. An onset entanglement concentration (c_e_) is necessary for fiber formation to occur. In the regime where c < c_e_, the polymer chains are far away from each other without sufficient contact and chain overlap, resulting in beads or beads with incipient fibers [67]. Beyond this onset concentration (c > c_e_), sufficient chain overlap causes a slight rise in viscosity and fibers with a beaded morphology (BOAS) are formed [71]. Above a critical concentration c *, continuous fibers are formed because a sufficient chain entanglement density is achieved that can inhibit jet breakup [72]. In Figure 4a, three regimes are identified for η_0_ ~ c with changing slopes (m) of 0.06, 0.23 and 0.40 between concentrations of 2–6 wt.%, 6–8 wt.% and 8–14 wt.%, respectively. Below a polymer concentration of 4 wt.%, very few fibers are formed, and a majority of beads is present due to insufficient chain overlap. An onset entanglement polymer concentration (c_e_) of 4 wt.% seems necessary to obtain sufficient chain overlap to form proper fibers. Although chain entanglement increases from 4 to 6 wt.% (as evidenced by a rise in the viscosity), it is still insufficient to form bead-free and continuous fibers. When the polymer concentration increases, a deformable entangled network of polymer chains forms because of sufficient chain overlap [73], as shown by a rise of the viscosity slope from m = 0.06 to m = 0.23. Starting from the critical transition region (c > c *) of approximately 8 wt.%, more continuous fibers are formed, and the slope of the viscosity curve further increases to m = 0.40. The changes in the slope of the viscosity curve (Figure 4a) are comparable to those of the median fiber diameter curve (Figure 4b), clearly showing the correlation between the polymer concentration, viscosity and fiber diameter. A polymer concentration of 10 wt.% seems effective to form continuous PHBHHx fibers with CFS (under the processing conditions used in the study). These trends correlate well with those of electrospun PHBHHx fibers, where bead-free and continuous fibers with increased diameters in a similar concentration range of 8–12 wt.% (chloroform solvent) were observed (PHBHHx M_w_ = 4.5 × 10^5^ g/mol) [25].

Lee at al. reported ES of continuous and bead-free PHB fibers at concentrations as low as 2 wt.%, while the required concentration for bead-free P(3HB-co-4HB) fibers was 5-fold higher (10 wt.%), and was attributed to the higher molecular weight of PHB [74]. The influence of molecular weight (and number of chain entanglements) indeed needs to be accounted for when comparing fiber morphology, diameter and properties. For example, Ol’khov et al. showed that not only an increase of the solution viscosity, but also an increase of molecular weight can result in more continuous electrospun PHB fibers with improved homogeneity [75]. Even the used carbon substrate for PHB synthesis can affect the fiber diameter and morphology [76].

Although previous studies showed a transition of beads to continuous electrospun PHA fibers with an increasing polymer concentration (solution viscosity) or molecular weight, the necessary concentration for the onset of continuous fiber production can differ extensively. For example, electrospun PHBV fibers showed a change to continuous fibers from 20 wt.% polymer concentration with a diameter range between 1.0–4.0 µm (M_w_ = 6.8 × 10^5^ g/mol) [77]. Yoon et al. reported the transition from BOAS to continuous electrospun PHBV fibers only from a concentration of 28 wt.% (M_w_ = 6.8 × 10^5^ g/mol) [22], while Tong et al. reported continuous electrospun PHBV fibers in a concentration range of 8–25 wt.% with fiber diameters of, respectively, 1.35 µm and 3.3 µm (M_w_ = 3.1 × 10^5^ g/mol) [78]. The latter explained that solutions with viscosity values in the range of 0.25–4 Pa·s may lead to the formation of continuous PHBV fibers, taking into account matched ES conditions [78]. Our reported solution viscosity values for CFS of continuous PHBHHx fibers (≥1.54 Pa·s) with average (${\overset{-}{D}}_{f}$) and median (${\tilde{D}}_{f}$) fiber diameters in the range of, respectively, 3.58–4.62 µm and 2.78–4.10 µm are comparable to those reported by [78]. Even though these values are similar, the properties, morphology and diameters still remain highly dependent on a combination of the used polymer (M_w_ and PDI) and processing conditions (collector distance, nozzle diameter, rpm, etc.), especially when comparing ES and CFS techniques.

### 3.3. Crystallization and Melting Behavior of PHBHHx Fibers

**Figure 5:**
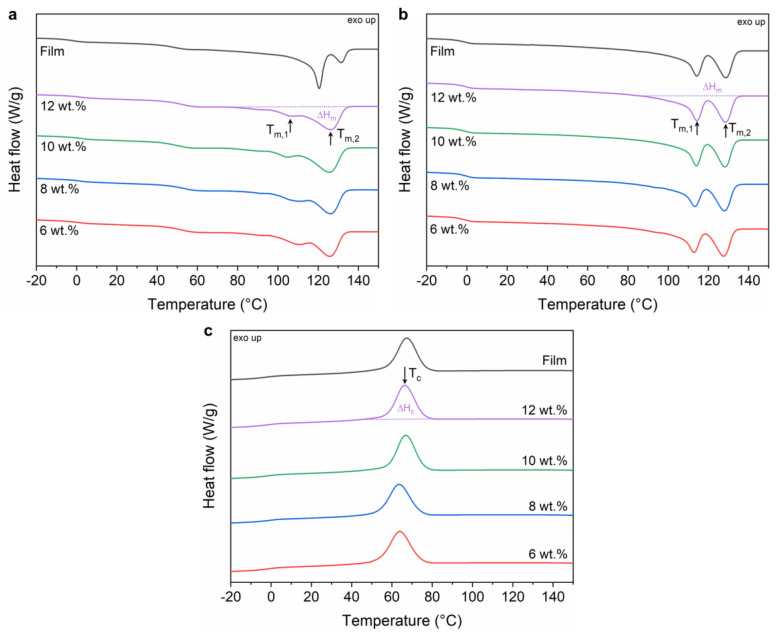
DSC thermograms of the first heating (**a**), second heating (**b**) and first cooling (**c**) cycles at a heating/cooling rate of 20 °C/min, representing the melting and crystallization behavior of PHBHHx fibers spun from 6, 8, 10, and 12 wt.% polymer/CHCl_3_ solutions and a compression molded PHBHHx film.

The melting and crystallization properties of PHBHHx fibers spun from 6, 8, 10 and 12 wt.% polymer concentrations are monitored from DSC analysis, as shown in Figure 5. The characteristic values are summarized in Table 1. For comparison, the respective data are shown for a compression molded film (fabricated from PHBHHx pellets as described in Section 2.4).

The first heating scan shows the thermal characteristics of the as-spun PHBHHx fibers. The multiple melting peaks are characteristic for PHBHHx materials [79], with a first major endothermic peak at T_m,1_ due to the melting of primary crystals formed during initial crystallization. Differences in the first major endothermic peak of the fibers are observed in the first heating cycle, with T_m,1_ ranging between 105.1–111.7 °C (Figure 5a). The first endothermic peak (T_m,1_) is broader at low polymer concentration (6–8 wt.%) whereas a reduction in peak melting temperature at higher polymer concentration (10–12 wt.%) is associated with a narrower and slightly more pronounced peak shape. These narrower melting peaks can indicate improved crystal perfection, while lower peak temperatures indicate a smaller spherulite size (lamella thickness) [80,81]. With increasing polymer concentration, restricted chain mobility can result in a decrease in spherulite size and size distribution [82], which can be correlated with a sharper endothermic melting peak apparent at a lower temperature. The second endothermic melting peak, T_m,2_ (~126 °C) is due to the melting of primary crystals formed during DSC heating [79,83]. No differences in T_m,2_ values are apparent for the different polymer concentrations. When compared with a PHBHHx film, both endothermic peaks are shifted to higher temperatures (T_m,1_ ~ 120 °C and T_m,2_ ~ 131 °C). In addition, the shape of the first melting peak is narrower with an increased peak area, indicating a larger crystal size with higher melting point. Further, the polymer film exhibits a decreased secondary peak area, indicating a less exhaustive recrystallization process during subsequent heating in DSC. These changes in melting peaks result in a higher crystallinity content for the bulk polymer film of ~37%, compared to ~33–34% for the fibers. The better crystallization of PHBHHx during compression molding into films with increased crystal sizes and narrow size distribution is explained by the allowed time to crystallize during the slow cooling process after molding (versus fast evaporation of CHCl_3_). In addition to the primary melting peaks, a minor endothermic transition at approximately 50 °C is also apparent in the first heating scan for both PHBHHx fibers and film, arising from a minority of imperfect crystals formed during storage at room temperature [84].

The second heating scan of the fibers (Figure 5b) also shows two major endothermic peaks (T_m,1_ and T_m,2_) at ~113–114 °C and ~128 °C, respectively. The size of the endothermic peak (at T_m,1_) increases compared to the first heating, indicating less recrystallization during subsequent heating because of the controlled cooling process during DSC. The melting enthalpy ΔH_m_ remains constant for all polymer concentrations but is slightly lower compared to the melting enthalpy of the first heating. In contrast to the first heating scan, the melting peak shapes and temperatures in the second heating scan are similar for the film and the fibers, due to the same thermal history induced by DSC cooling. The glass transition occurs around −1 °C to 0 °C for both fibers and film.

In comparison with literature data on different processing routes for PHBHHx, the size of the primary melting peak (at T_m,1_) of CFS fibers during the first heating scan is smaller and less pronounced and the crystallinity content is lower compared to those of extrusion and injection molded PHBHHx (X_c_ = ~38%) [11], which indicates a less developed structure in the fibers. These changes are similar to those of a compression molded PHBHHx film (Table 1). A possible explanation is that the rapid arrangement of stretched chains under large elongational strains after solvent evaporation hinders the crystallization process during spinning [62]. The polymer molecules have less time to realign themselves due to rapid solvent evaporation and jet temperature reduction, leading to less favorable packing and a less developed crystal structure [85]. The temperature of the secondary melting peak (T_m,2_ ~ 126 °C) in CFS fibers occurs around the same temperature as extrusion and injection molded PHBHHx, which, however, changes slightly depending on the mold temperature (T_m,2_ ~ 126–131 °C) [11].

A comparison of the cooling curves in Figure 5c shows that the crystallization peak T_c_ arises at ~63 °C for 6 and 8 wt.% polymer concentrations, but increases to >66 °C, at higher polymer concentrations (10 and 12 wt.%). During cooling, the crystallization enthalpy (ΔH_c_) slightly increases from 33.6 to 34.1 J/g. The crystallization peak temperature and enthalpy of the PHBHHx film are similar to those of fibers spun from 10–12 wt.% concentrations (Table 1).

Despite some changes in the first endothermic melting peak of the as-spun PHBHHx fibers from different concentrations, no severe changes in crystallinity content are apparent. In addition, the small changes in crystallinity (33.0–34.3%) are independent of the PHBHHx concentration. Similar to our results, it was found that the crystallinity content (61–65%) and melting temperature (173.2–174.0 °C) of electrospun PHB fibers remains relatively constant and independent of the PHB concentration (6–13 wt.%) [86]. Other reports on electrospun PHB fibers showed that the crystalline phase of PHB was not altered by changing the spinning conditions and a similar crystallinity degree (53%) compared to solvent casted films was obtained [87]. In line with our results, Mottin et al. obtained electrospun PHB fibers with lower crystallinity (as evidenced by FTIR and WAXD) compared to compression molded films [88]. Similarly, a lower melting enthalpy (and lower crystallinity) was also reported for electrospun PHB [89], PHBHHx [24] and PHBV [90,91], and centrifugally spun PHBV [48], compared to their bulk counterparts.

Our findings are in contrast with a CFS study on poly(ethylene oxide) (PEO), where an increase in polymer concentration resulted in higher fiber crystallinity content [63]. However, this trend was not further elucidated. Others also reported the increased crystallinity of electrospun PHB fibers with increasing polymer concentration and explained this by the shielding of PHB polymer chains from the solvent, thus remaining more ordered in solution prior to spinning [89]. On the other hand, in the case of centrifugally spun polyacrylonitrile (PAN) fibers, the crystallinity decreased with increasing polymer concentration owing to increased polymer chain entanglements [45], as well as in the case of electrospun PLLA, where the decrease in polymer crystallinity at higher polymer concentrations was ascribed to the reduced molecular orientation during spinning (due to higher solution viscosity) [92]. Additionally, the collection method of electrospun PHBHHx fibers can clearly influence the crystal structure and molecular chains in the crystals [29]. In summary, the relatively constant crystallinity of centrifugally spun PHBHHx can be compared to some previously reported studies on PHA fibers. However, the crystallinity and crystal structure of both electro- and centrifugally spun fibers seem highly dependent on polymer properties (type, M_w_), processing conditions and fiber collection method.

### 3.4. Mechanical Properties of PHBHHx Fiber Mats

**Figure 6:**
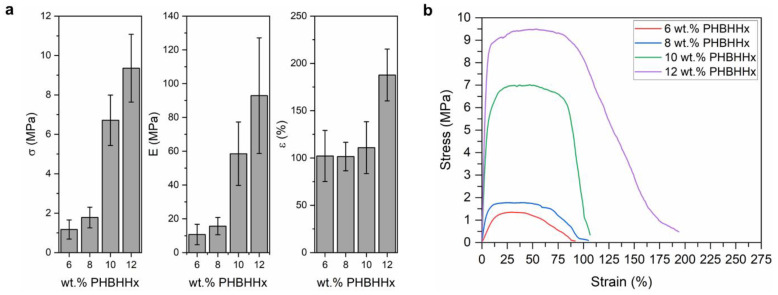
(**a**) Tensile strength (σ), Young’s modulus (E) and elongation at break (ε) (*n* = 10, error bars represent SD), and (**b**) representative stress-strain curves of fiber mats spun from 6, 8, 10, and 12 wt.% polymer/CHCl_3_ solutions.

The mechanical properties of the collected PHBHHx fiber mats and representative stress-strain curves are shown in Figure 6. The stress-strain data include the variation of the fiber mat weight (as explained in Section 2.3.4). The average tensile strength, Young’s modulus and elongation at break of the PHBHHx fiber mats vary between 1.2 MPa, 11 MPa and 102% for fiber mats made from 6 wt.% polymer solutions to 9.4 MPa, 93 MPa and 188% for fiber mats made from 12 wt.% polymer solutions (Figure 6a). The strength and stiffness of the fiber mats increases with increasing polymer concentration, particularly at concentrations of 10 and 12 wt.% PHBHHx. This can be attributed to both the changes in fiber diameter and morphology of the fiber surface. First, the highest increase in tensile strength and Young’s modulus occurs between polymer concentrations of 8 and 10 wt.%, similar to the steep increase of the fiber diameter with increasing polymer concentration (Figure 4b). In this way, the trends for strength and stiffness of the fiber mats correlate well with those of the solution viscosity and fiber diameter. On the other hand, the increased strength of the fiber mats can also be explained by the fact that smooth and continuous fibers (shown in Figure 2) have more fiber cohesion points (interaction points) and fewer beads acting as fiber defects [93]. The elongation at break of the fiber mats is around 100% for polymer concentrations between 6–10 wt.% but increases to values of 188 ± 27% at 12 wt.% PHBHHx. The observed higher elongation at a break of 12 wt.% fibers can be explained by the fact that these fibers are thicker, very smooth, continuous and bead-free. Some occasional bead formation can still be present in the 10 wt.% fibers and it is known that beads on the surface of the fibers can reduce the cohesive force between the fibers of the mat, resulting in poorer mechanical properties such as a reduced elongation at break [94,95]. Therefore, finer fibers spun from low concentration solutions have reduced elongation at break (and lower strength) because they show more bead formation [96].

The tensile data of the obtained PHBHHx fiber mats show relatively high standard deviations (SD), which was also reported for centrifugally spun PHBV and was explained by the stochastic nature of testing nonwoven mats [48]. In addition, a rather broad fiber diameter distribution and the variation in fiber packing/density of centrifugally spun mats can account for the relative high variability in mechanical properties.

The observed trend of higher σ, E and ε at higher polymer concentrations was also associated with a larger diameter and more perfect fibers of electrospun PET fibers [97]. Similar to our reported mechanical properties, a steep increase in elongation at break (more than twofold), from 4.5 to 10.6%, was also observed for electrospun PHB when the fiber diameter was increased from 0.45 to 3.14 µm [28]. However, this increase in elongation was associated with a decrease in both tensile strength and Young’s modulus. To further compare with PHA fibers, σ, E and ε values of electrospun PHBHHx meshes fabricated from a solution concentration of 15 w/v% were previously reported to be around 2.3 MPa, 66 MPa and 61% for randomly oriented meshes and 4.6 MPa, 156 MPa and 7% for aligned meshes, respectively [26]. The stronger aligned meshes also exhibited larger fiber diameters compared to the randomly aligned ones. These tensile properties were measured at the same speed of 10 mm/min and fiber mat size of 100 mm × 10 mm. Centrifugally spun PHBV fibers showed average tensile strength and Young’s modulus values of, respectively, 3 MPa and 100 MPa for PHBV fibers produced at a speed of 9000 rpm (20–25 *w*/*v*%) [48]. The strength and stiffness of the PHBV fibers also increased with fiber orientation. These studies show that the collection method and processing conditions of fiber mats can influence the mechanical properties because of changes in fiber mat orientation (aligned vs. randomly oriented). Borisova et al. showed that the induced alignment of the fibers, by changing collector type and speed, can increase the mechanical properties and crystallinity of electrospun PHB [98]. They showed that tensile testing of more oriented fiber mats in the direction of alignment results in enhanced mechanical properties, compared to testing in the perpendicular direction [98]. In addition, Volova et al. showed that the effect of fiber orientation on the physical-mechanical properties of electrospun PHAs is the strongest, followed by the PHA chemical composition (HHx, HV, 4HB) and polymer concentration [28]. Therefore, besides an increased fiber diameter and bead reduction, the slightly increased fiber orientation at a higher polymer concentration (due to a higher production rate) could also contribute to the observed enhanced tensile strength and Young’s modulus of PHBHHx fiber mats at higher concentrations (10–12 wt.%).

In addition, the tensile strength and elongation at break of electrospun PHB were reported to be ~48.5 MPa and ~94% for fiber diameters of 0.676 ± 0.083 µm (speed 10 mm/min, sample 100 mm × 10 mm) [99]. Fernandes et al. reported σ, E and ε values for electrospun PHB of, respectively, ~0.95 MPa, ~54 MPa and ~10% (tensile speed 0.5 mm/min, sample 40 mm × 5 mm × 40 µm) [100]. In this way, comparing the mechanical properties of fiber mats still remains challenging, because they often differ in fiber diameter, sample size and the tensile test procedure applied. For example, a higher packing density of the fiber mat can also exhibit better mechanical properties because of the higher generated force resisting fiber reorientation during tensile drawing [101]. Rashid et al. reviewed the challenges in comparing mechanical properties of fibers and fiber mats [102]. Some challenges included the absence of a standardized testing protocol and the influence of fiber diameter and collector type. To conclude, the mechanical properties of PHBHHx fibers by CFS are comparable to those of electro- and centrifugally spun PHA fiber mats and it was identified in this work that the mechanical properties mainly depend on the fiber diameter, bead defects, mat density/porosity and fiber entanglement.

### 3.5. Application Potential of Centrifugally Spun PHBHHx Fibers to Form Films

**Figure 7:**
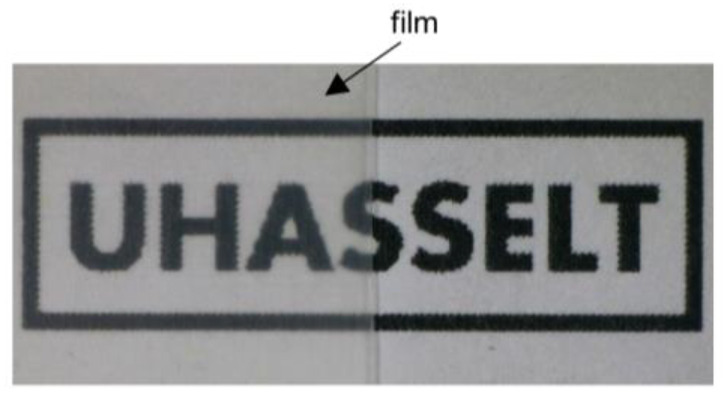
Picture of a PHBHHx film composed of two attached layers: a PHBHHx substrate and an annealed top-layer of centrifugally spun PHBHHx fibers from 10 wt.% polymer/CHCl_3_ solutions.

As a proof-of-concept, fiber mats (~1 g) were heat-treated at 145 °C under pressure (hot-press) to form films of ±100 µm thickness by annealing the fibers into continuous and translucent layers. The fibers were spun from 10 wt.% PHBHHx/CHCl_3_ solutions since they show a continuous fiber morphology and can be produced at a relative high production rate. In order to further explore the application potential of centrifugally spun PHBHHx fibers, lower amounts of thin fiber mats were deposited as a top-layer on a PHBHHx substrate by thermal posttreatment (Figure 7). For the top-layer deposition, annealing for at least 6 min at 160 °C was required to form a continuous layer on the PHBHHx substrate film. Higher temperatures (to decrease the annealing time) resulted in the undesired melting of the PHBHHx substrate. Therefore, the optimization of the annealing time and temperature (to promote the adhesion between top-layer and substrate) is closely associated with the thermal properties of the used fiber and substrate materials. Still, the use of higher temperatures should be limited because the PHBHHx material can be thermally degraded under the influence of increased temperatures [11,103].

Annealing times of 5 min at 160 °C have also resulted in compact and continuous PHB films (electrospinning) [34]. In contrast, annealing times as short as 5 s have been suggested to produce films and multilayer structures of PHB and PHBV [35,36,40]. A possible explanation for the higher annealing time in this study could be the fact that the aluminum plates and Teflon sheets limit the transfer of heat from the hot-press plates to the fibers during the annealing process. The top-layer film shows suitable transparency and has a homogenous surface appearance, as shown in Figure 7. The top-layer does not affect the transparent nature of the substrate, which is often desired in specific applications, e.g., packaging.

**Figure 8:**
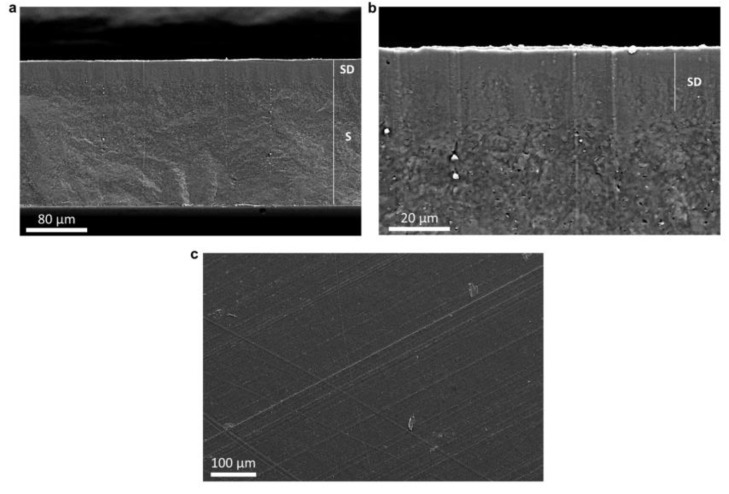
SEM images of the cross sections (**a**,**b**) and surface (**c**) of PHBHHx films fabricated by deposition of 10 wt.% polymer/CHCl_3_ solution spun PHBHHx fibers onto PHBHHx substrates. The cross section is marked with the upper surface deposition layer (SD) and the PHBHHx substrate (S).

According to morphological SEM analysis, the produced top-layer films clearly exhibit a continuous surface deposition layer (SD), originating from the PHBHHx fibers, and a substrate layer (S) (Figure 8a,b). The SD layer is strongly adhered to the substrate with little to no porosity (qualitatively determined from SEM), indicating a proper annealing process for the production of continuous films. The thickness of the SD layer is around 10–20 µm and can be slightly variable from sample to sample due to possible variations in the manual collection of fiber mats during the CFS process. In addition, variations in the top-layer thickness are expected since the material is fabricated by the coalescence of fiber mats with a variable porosity and packing density. Therefore, the thickness of the deposition layer depends on the amount of collected fibers and mat porosity/density, which is tunable during the CFS process to a certain extent. The thickness of the substrate layer is defined by the mold design (160–170 µm), which is customizable. The surface of the films (Figure 8c) is continuous, and no clear traces of fibers remain after the annealing treatment. The lines on the surface originate from the Teflon sheets used during annealing in the hot press.

The developed PHBHHx films fabricated using CFS are suitable for a range of applications as they provide a unique method to introduce specific functionality. For example, Cherpinski et al. showed that annealed biopolymer coatings (including PHB) can improve the water, aroma and oxygen barrier of fiber-based substrates depending on the coating thickness [39]. In addition, multilayer structures can be fabricated with active ingredients (e.g., inorganic nanoparticles) in the top-layer to add a UV barrier, a gas barrier or antimicrobial activity to the material. Applications with electrospun PHAs (with fillers) as top-layers on a range of substrates have been reported previously. Electrospun multilayer structures of PHBV with silver [104], copper oxide [105] and zinc oxide [106] nanoparticles showed a suitable dispersion quality and antimicrobial activity.

## 4. Conclusions

This study shows the production of PHBHHx fibers by centrifugal fiber spinning of chloroform solutions with different polymer concentrations (4–12 wt.%) and the post-processing into continuous films and top-layers. To the best of our knowledge, this has not been reported before. The results reveal the transition from a beads-on-a-string morphology in the low polymer concentration domain (≤8 wt.%) to a continuous fiber morphology with few beads in the higher polymer concentration domain (≥10 wt.%). This morphological transition results from a significant increase in solution viscosity and is associated with a corresponding increase in fiber diameter (${\overset{-}{D}}_{f}$ = 0.5–4.6 µm). The continuous fibers produced from higher polymer concentrations (10–12 wt.%) show enhanced mechanical properties, such as strength and stiffness, which is attributed to the increased fiber thickness and reduction of beaded defects. The fiber crystallinity is independent of the polymer concentration and remains relatively constant, while only minor changes in crystal structure are apparent. The trends in the morphological, mechanical and thermal properties of PHBHHx are comparable to those of electrospun and centrifugally spun PHA fibers, previously reported in literature. However, we clearly highlight the challenges in comparing mechanical and thermal properties of fiber mats, because they often differ in polymer properties (M_w_), fiber diameter, mat porosity/density/size, processing and collection method and the characterization procedure applied. Further, we show that the loosely packed PHBHHx fiber mats produced by CFS can be thermally post-treated into continuous films and compact top-layers of about 10–20 µm. The development of such materials could have great potential for use in an industrial context because of the higher production rate and scalability compared to ES.

The centrifugally spun PHBHHx fiber mats with sufficient strength and flexibility show great potential and can be tailored to meet a wide variety of applications, including drug delivery, tissue engineering etc. These fiber mats could also be post-processed and applied as water vapor barrier layers on fiber based substrates [39]. In addition, future research can focus on the incorporation of active ingredients such as inorganic nanoparticles in the PHBHHx fibers with high dispersion quality in order to develop active packaging applications (UV barrier, antimicrobial).

## Figures and Tables

**Table 1:** Thermal characteristics of the centrifugally spun PHBHHx fibers (6, 8, 10 and 12 wt.% polymer/CHCl_3_ solutions) and compression molded PHBHHx film for DSC 1st heating, 1st cooling and 2nd heating cycles determined at a heating/cooling rate of 20 °C/min (*n* = 2, ±1 SD). Melting peaks are denoted as T_m,1_ and T_m,2_, crystallinity content as X_c_, crystallization peak temperature as T_c,p_, and melting and crystallization enthalpies as ΔH_m_ and ΔH_c_, respectively.

**PHBHHx Sample**	**1st Heating**					**2nd Heating**			
	**T_m,1_ (°C)**	**T_m,2_ (°C)**		**ΔH_m_ (J/g)**	**X_c_ (%)**	**T_m,1_ (°C)**		**T_m,2_ (°C)**	**ΔH_m_ (J/g)**
6 wt.%	111.1 ± 0.0	125.9 ± 0.0		38.4 ± 1.0	33.4 ± 0.9	113.0 ± 0.1		127.7 ± 0.2	36.9 ± 0.9
8 wt.%	111.7 ± 0.2	126.3 ± 0.2		38.5 ± 1.4	33.5 ± 1.3	113.4 ± 0.2		128.1 ± 0.2	36.5 ± 0.5
10 wt.%	105.1 ± 0.6	125.6 ± 0.1		39.4 ± 0.0	34.3 ± 0.0	114.1 ± 0.0		128.3 ± 0.1	37.0 ± 0.5
12 wt.%	107.0 ± 0.5	126.2 ± 0.1		38.0 ± 0.5	33.0 ± 0.4	114.4 ± 0.2		128.7 ± 0.2	35.9 ± 0.4
Film	120.1 ± 0.7	131.3 ± 0.3		42.3 ± 0.5	36.7 ± 0.4	114.1 ± 0.2		128.6 ± 0.0	37.4 ± 0.0
**PHBHHx** **Sample**			**1st Cooling**						
			**T_c,p_ (°C)**				**ΔH_c_ (J/g)**		
6 wt.%			63.9 ± 0.1				33.6 ± 0.5		
8 wt.%			63.6 ± 0.0				33.8 ± 0.7		
10 wt.%			66.9 ± 0.0				34.1 ± 0.1		
12 wt.%			66.3 ± 0.1				33.7 ± 0.4		
Film			67.1 ± 0.4				33.5 ± 0.4		

## Data Availability

Data is contained within the article.
